# Taking a long look at isochrony: Perceived duration increases with temporal, but not stimulus regularity

**DOI:** 10.3758/s13414-014-0787-z

**Published:** 2014-10-24

**Authors:** Ninja K. Horr, Massimiliano Di Luca

**Affiliations:** Centre for Computational Neuroscience and Cognitive Robotics, Department of Psychology, University of Birmingham, Edgbaston, Birmingham, B15 2TT UK

**Keywords:** Temporal perception, Perceived duration, Filled-duration illusion, Isochrony, Entrainment

## Abstract

A commonly observed phenomenon to elucidate distortions of perceived duration is the filled-duration illusion: a temporal interval delimited by two marker signals is perceived to be shorter than the same interval with several identical filler signals. Previous investigations have focused on regularly spaced (isochronous) fillers and the influence of their temporal structure has not been considered. We find that intervals with isochronous fillers are perceived to last longer than their anisochronous counterparts. The illusion increases with the amount of deviation from isochrony and with the number of fillers. Findings also indicate that perceived duration is specifically affected by temporal irregularities, as randomization of the fillers’ sound amplitude or frequency does not cause an appreciable distortion. These results can be accounted for by both pacemaker-accumulator models and entrainment models.

## Introduction

Stimulus duration is not always perceived veridically, because it depends on many factors beyond physical time (see Allan, 1979 for a classic and Grondin, 2010 for a recent review). For example, nontemporal stimulus characteristics, such as familiarity (e.g., Devane, 1974; Witherspoon & Allan, 1985), complexity (e.g., Schiffman, & Bobko, 1974), sensory modality (e.g., Goldstone & Lhamon, 1974; Wearden, Todd & Jones, 2006), and context (e.g., Dyjas & Ulrich, 2013; Hellström, 2003), influence perceived interval duration. Disentangling the principles and mechanisms underlying such effects is crucial for the development of a realistic model of temporal perception.

A striking source of distortions in perceived duration is due to the filling of the interval to be judged. A long-known phenomenon, which has been replicated with several experimental variations, is the filled-duration illusion whereby filled intervals are perceived to last longer than empty intervals of the same duration. Empty intervals in this context can be intervals defined solely by a beginning and an end marker (e.g., Rammsayer & Lima, 1991), but also can be implemented as a gap in an otherwise continuous signal (e.g., Rammsayer & Leutner, 1996; Wearden, Norten, Mayer, & Oliver, 2007). Filled intervals, instead, can be continuous signals (e.g., Hasuo, Nakajima, Tomimatsu, Grondin, & Ueda, 2014; Rammsayer & Lima, 1991) or intervals consisting of a number of regularly spaced fillers (e.g., Adams, 1977; Buffardi, 1971; Thomas & Brown, 1974).

In comparison to the multitude of studies addressing the filled-duration illusion, there is surprisingly little research investigating whether and how filler characteristics and temporal structure influence duration judgments. One of the few exceptions are findings showing that perceived duration increases with the number of fillers and that fillers presented towards the beginning of the interval lead to longer perceived duration than fillers presented towards the end (e.g., Adams, 1977; Buffardi, 1971; Goldstone & Goldfarb, 1963; Schiffman & Bobko, 1977). Furthermore, Grimm (1934) asked participants to compare regularly and irregularly spaced intervals of the same physical duration and found that regularly spaced intervals are more frequently judged as longer in a three alternative task (longer, shorter, or equal). Using a temporal reproduction task, Thomas and Brown (1974) failed to observe a significant difference in perceived duration between regular and irregular intervals, although there were more responses indicating shorter irregular stimuli. Matthews (2013) recently reported how regularly spaced fillers are perceived longer than accelerating or decelerating ones. These results suggest that the timing of the fillers can play an important role in the estimation of interval duration.

We investigated whether deviations from isochrony and filler regularity lead to distortions of perceived duration. All experiments employed a duration discrimination task in which participants judged which of two intervals appeared to last longer (two-interval forced choice, 2IFC). This allowed to increase measurement sensitivity and to diminish response biases that could have affected early results (e.g., Thomas and Brown, 1974) to quantify the magnitude of the effect. Each trial comprised two intervals: one with isochronous auditory beeps and one where the timing of beeps diverged from isochrony (the order of the two types of intervals was random and counterbalanced). Either of the two intervals varied in duration across trials; that is, we varied the time between the beginning of the first beep to the ending of the last and all of the segments accordingly. In Experiment 1, we investigated whether the amount of variation in the regularity of fillers influences duration perception. In Experiment 2, we tested the influence of filler density (the number of fillers in a fixed time) on the observed effect of temporal structure. Experiment 3 served to find out whether irregularity of nontemporal filler properties (sound amplitude or frequency) could also influence perceived duration.

## General methods

### Participants

A total of 64 students from the University of Birmingham participated in the experiments for course credits or a payment of 6 GBP/h. Participants were naive to the purpose of the investigation, reported normal auditory sensitivity, and took part in only one of the experiments. Experimental procedure and data collection followed the ethical guidelines of the Declaration of Helsinki (2012) and was approved by the Science, Technology, Engineering & Mathematics Ethical Review Committee of the University of Birmingham.

### Experimental design

**Fig. 1:**
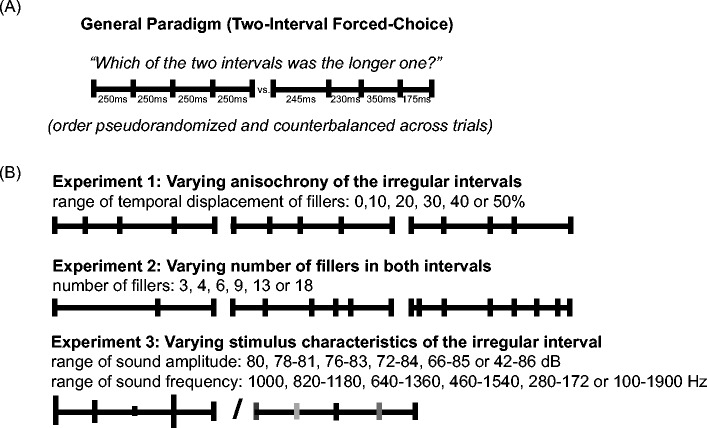
Overview of the 2IFC tasks in the experiments. **a** General paradigm: in each trial participants compare the duration between two intervals (one regular and one irregular, order randomized). **b** Experiment 1 (top): different levels of anisochrony are presented (and compared against isochrony). Experiment 2 (middle): different numbers of fillers are presented (equal for the two, one isochronous, one anisochronous, intervals to be compared). Experiment 3 (bottom): two isochronous intervals are presented, one regular, one irregular, in the irregular interval fillers vary in sound amplitude (Experiment 3a) or sound frequency (Experiment 3b)

Participants reported which of two intervals appeared to last longer (2IFC, Fig. 1a). One interval was regular and one was irregular (in Experiments 1 and 2 the regular interval was isochronous and the irregular interval was anisochronous; in Experiment 3a and 3b both the regular and the irregular interval were isochronous, but the fillers of the irregular interval had varying properties). One of the intervals was always 1000 ms (standard); the other one (counterbalanced between the regular and the irregular interval) could be 500, 700, 850, 1000, 1150, 1300, or 1500 ms (comparison). The order of regular and irregular as well as of standard and comparison intervals was random and counterbalanced. The proportion of regular intervals reported to be longer than irregular intervals were obtained at each level of duration difference between regular and irregular. The points of subjective equality (PSE) and the just noticeable differences (JND) were estimated using the Spearman-Kärber Method as the first and second moments of the distribution (Ulrich & Miller, 2004).

PSE values represent the physical duration difference between the regular and the irregular interval at which perceived duration is equal (in milliseconds). A positive PSE value indicates the overestimation of the irregular interval. A negative PSE value indicates its underestimation. JND values indicate the duration difference at which subjects can discriminate the duration of the two intervals (again in milliseconds). The fillers making up the intervals were 10 ms tones (1000 Hz in Experiments 1 and 2) with 1-ms onset and offset tapering. A gap of 3 seconds separated the presentation of the two intervals to be compared. An overview of the conditions tested in the 3 experiments is given in Fig. 1. All experiments lasted approximately 1 hour.

## Experiment 1

To investigate whether and how the temporal structure of fillers influences perceived duration, we asked participant to compare isochronous sequences of fillers to anisochronous sequences and varied the level of anisochrony in the irregularly spaced interval (Fig. 1b, top).

### Material and methods

Twenty students (15 female, mean age =21.0 ± 4.2) participated in the experiment. Intervals contained five fillers (10 ms, 1000 Hz, 70 dB SPL tones). Trials consisted of one isochronous and one anisochronous interval. The anisochronous intervals were created by randomizing the time of the three middle filler signals. The time at which fillers were presented was perturbed by randomly sampling from a uniform distribution of ±10, 20, 30, 40 or 50 % of the duration of the otherwise constant interstimulus interval (ISI). For the 1000 ms standard interval, the ISI corresponded to a jitter that could reach ±25, 50, 75, 100, 125 ms respectively. It should be noted that randomization by 50 % of the ISI is the highest anisochrony that prevents two successive fillers to overlap. Participants performed 336 duration discrimination judgments resulting from 8 repetitions of 42 trials obtained through all combinations of comparison duration (7) and levels of anisochrony (6).

### Results and discussion

**Fig. 2:**
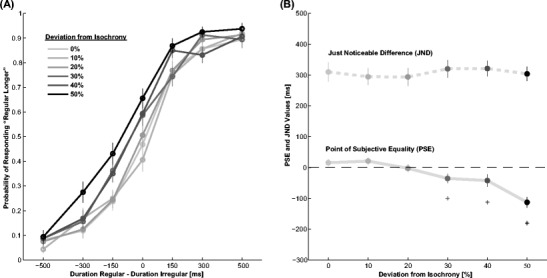
Results of Experiment 1. **a** Probability of the isochronous interval being reported as longer over the physical difference between isochronous and anisochronous interval duration. **b** PSE and JND values for the different levels of anisochrony. Asterisks indicate a significant difference to the zero deviation from isochrony PSE (*p* <0.05). Error bars are S.E.M.

From the proportion of responses as a function of the difference in physical duration between the regular and the irregular interval (Fig. 2a), we obtained PSE and JND values for each level of anisochrony (Fig. 2b). Visual inspection hints at a decrease of the PSE with an increase in the level of anisochrony. Due to the frequently observed influence of stimulus order on duration judgments (e.g., Allan, 1977; Dyjas & Ulrich, 2013; Hellström, 2003) and the idea that the presentation of a regular sequence might influence duration perception of following intervals (e.g., Halpern & Darwin, 1982; McAuley & Jones, 2003), we also included the order of isochronous and anisochronous intervals into our statistical analysis by calculating PSEs separately for isochronous first and anisochronous first trials.

A two-way repeated measure ANOVA on PSE values with factors level of anisochrony (0, 10, 20, 30, 40, or 50 %) and order of intervals (regular first or irregular first) was conducted. The difference in duration between regular and irregular intervals increases with the level of anisochrony (Fig. 2a) as revealed by the significant main effect of anisochrony on PSE values (*F*(5,95) = 9.3, *p* <0.001, *η*_*p*_^2^ = 0.33). Post-hoc tests reveal a significantly longer perceived duration of the isochronous interval for conditions with anisochrony >30 % (single sample *t*-test on PSE against zero asynchrony two-tailed, significant outcomes are reported as asterisks in Fig. 2b: 10 %, *t*(19) = 0.3, *p* = 0.76; 20 %, *t*(19) = −1.2, *p* = 0.27; 30 %, *t*(19) = −2.4, *p* = 0.026; 40 %, *t*(19) = −2.7, *p* = 0.014; 50 %, *t*(19) = −5.8, *p* <0.001). Comparing PSE values of adjacent conditions there is a close to significant decrease of PSE values between 20 and 30 %, the major significant decrease takes place between 40 and 50 % asynchrony (10 vs. 20 %, *t*(19) = 1.4, *p* = 0.18; 20 vs. 30 %, *t*(19) = 1.8, *p* = 0.09; 30 vs. 40 %, *t*(19) = 0.3, *p* = 0.75; 40 vs. 50 %, *t*(19) = 3.5, *p* = 0.003).

As shown by a main effect of interval order, irregular intervals are perceived to be shorter when they are presented first in the trial than when they are presented second with a difference of 52 ms ±16 ms (mean ±standard error of the mean [SEM]; *F*(1,19) = 11.0, *p* = 0.004, *η*_*p*_^*2*^ = 0.37). The significant effect of interval order is in accordance with the frequent observation that the first interval in a discrimination task is being perceived as shorter than the second one (e.g., Allan, 1977; Hellström, 2003). The interaction of the two factors (level of anisochrony and interval order) is not significant (NS; *F*(5,95) = 1.4). This lack of an interaction shows that the bias toward underestimating the first interval is independent of the effect of judging isochronous intervals as longer than anisochronous intervals.

An overall reasonable performance is indicated by the mean JND value of 307 ms ±23 ms. The order of presentation of regular and irregular intervals affects performance (*F*(1,19) = 15.7, *p* = 0.001, *η*_*p*_^*2*^ = 0.45; two-way repeated measurement ANOVA of JND values with factors interval order and level of anisochrony) with performance being worse if the isochronous interval is presented first (310 ms ±24 ms vs. 255 ms ±26 ms). The level of anisochrony does not affect duration comparison performance (*F*(5,95) = 0.6, NS) and neither does so in conjunction with order (*F*(5,95) = 1.9, NS).

In sum, the results of Experiment 1 indicate that the temporal structure of fillers has a strong influence on perceived duration. Specifically, isochronous spacing of fillers leads to longer perceived duration compared with anisochronous spacing and the difference increases with the level of anisochrony. The effect could be observed independent of the temporal order of isochronous and anisochronous intervals. A question that remains open from Experiment 1 is to what extend the effect depends on the rate at which filler stimuli are presented, that is, the number of fillers in the one second standard interval.

## Experiment 2

Experiment 2 investigated whether the difference in perceived duration between isochronous and anisochronous intervals is modulated by the presentation rate for filler signals (Fig. 1b, middle). We tested this by increasing the number of fillers in the interval while maintaining the average duration of the intervals (1 second), thus affecting the density of the interval and the number of fillers per second.

### Material and methods

Twenty students participated in the experiment (18 females, mean age =19.6 ±1.4). The fillers in the irregular interval were spaced according to the highest level of anisochrony used in Experiment 1 (in a range of 50 % of ISI). In every trial, two intervals with an equal number of fillers were compared. The average duration of all intervals was 1 second. There were 6 blocks where the intervals were made of 3, 4, 6, 9, 13, or 18 fillers. Each block comprised 56 trials, resulting from 8 repetitions of the 7 comparison durations.

### Results and discussion

**Fig. 3:**
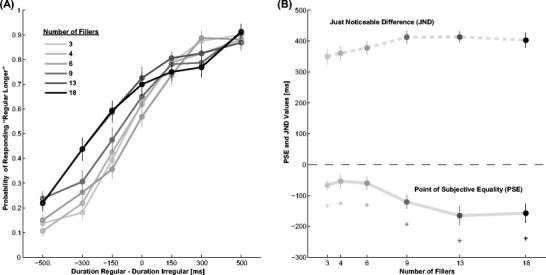
Results of Experiment 2. **a** Probability of the isochronous interval being reported as longer over the physical difference between isochronous and anisochronous interval duration. **b** PSE and JND values for the different numbers of fillers. Asterisks indicate a significant difference to zero (*p* <0.05). Error bars are SEM

Results are displayed in Fig. 3, and they replicate the findings of Experiment 1. Isochronous intervals are perceived to be longer than their anisochronous counterparts. The effect is present with every number of fillers tested (3, *t*(19) = −4.1, *p* <0.001; 4, *t*(19) = −2.5, *p* = 0.022; 6, *t*(19) = −2.8, *p* = 0.011; 9, *t*(19) = −5.3, *p* <0.001; 13, *t*(19) = −5.3, *p* <0.001; 18, *t*(19) = −4.9, *p* <0.001), even though the effect measured in ms gets stronger as a function of the number of fillers.

In a two-way repeated measurement ANOVA on PSE values a main effect of number of stimuli is observed (*F*(5,95) = 4.8, *p* <0.001, *η*_*p*_^*2*^ = 0.20). Post-hoc tests reveal that a significant decrease of PSE takes place between 6 and 9 filler stimuli (3 vs. 4: *t*(19) = −0.6, *p* = 0.54; 4 vs. 6: *t*(19) =0.3, *p* = 0.77; 6 vs. 9: *t*(19) =2.3, *p* = 0.032; 9 vs. 13, *t*(19) =1.2, *p* = 0.23, 13 vs. 18, *t*(19) = −0.2, *p* = 0.84).

Interval order is influencing the judgment in the same direction as in Experiment 1; that is, the irregular interval is perceived as shorter when it is presented first compared with when it is presented second (*F*(1,19) = 25.4, *p* <0.001, *η*_*p*_^*2*^ = 0.57), and the interaction with filler number is not significant (F(5,94) = 1.5, n.s). The overall mean JND is 386 ms ±16 ms. No significant effects have been found on JNDs (number of fillers: *F*(5,95) =1.7, NS; stimulus order: *F*(1,19) =0.1, NS; interaction: *F*(5,95) =1.4, NS).

In sum, isochronous intervals are perceived to be longer than anisochronous ones over a wide range of filler rates. The difference in perceived duration increases with more fillers.

## Experiment 3

Experiment 3 was conducted to test whether the observed effect of temporal structure can be generalized to nontemporal irregularities in filler characteristics (Fig. 1b, bottom). Therefore, the independent variable was the level of irregularity of the fillers regarding sound amplitude (Experiment 3a) or sound frequency (Experiment 3b).

### Material and methods

Seventeen students (all female, mean age =19.1 ±0.8) participated in Experiment 3a and another 17 students (15 females, mean age =19.5 ±1.0) participated in Experiment 3b. Both intervals presented in a trial were now regularly spaced (isochronous) and contained five fillers. For the regular interval, the fillers were identical (1000 Hz, 80 dB SPL), whereas for the irregular interval they varied at random in either their acoustic amplitude (Experiment 3a) or frequency (Experiment 3b). There were six levels of amplitude and frequency variation. Amplitudes varied in a range of either 80 ±0, 78.1–81.7, 75.6–82.9, 72.2–84.0, 66.4–85.1, or 41.9–86.0 dB SPL. Sound frequencies varied approximately 1000 Hz in a range of ±0, ±180, ±360, ±540, ±720, or ±900 Hz. Due to sound amplitudes up to 86 dB, stimuli were, in contrast to Experiment 1 and 2, presented via speakers. As in Experiment 1, the independent variable was varied in a trial-by-trial fashion, so that there were 8 blocks of 42 trials each (7 durations of the standard stimulus times 6 ranges of variation).

### Results and discussion

**Fig. 4:**
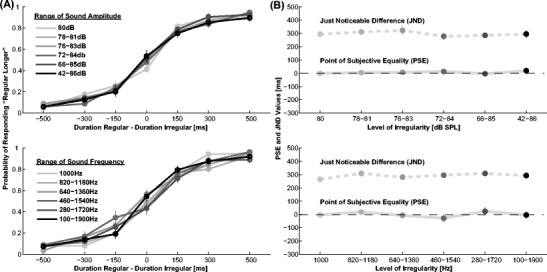
Results of Experiment 3. **a** Probability of the regular interval being reported as longer over the physical difference between regular and irregular interval duration for Experiment 3a (top) and Experiment 3b (bottom). **b** PSE and JND values for the different levels of irregularity, that is, the different ranges of sound amplitude (top) and sound frequency (bottom). Error bars are SEM

Figure 4 shows the response proportions as well as PSE and JND values for Experiment 3a and 3b. As expected from visual inspection, there is no significant change in perceived duration due to increased amplitude irregularity (2-way repeated measurement ANOVA on PSE, *F*(5,80) =0.4, NS) nor to sound frequency (*F*(5, 80) =0.9, NS). The effect of interval order as well was not significant in Experiment 3a (*F*(1,16) =0.7, NS) and there was no interaction (*F*(5,80) =1.0, n.s).

There was no interaction between irregularity and interval order (*F*(5,80) =0.6, NS). The overall mean JND was 297 ms ±19 ms in Experiment 3a and 292 ms ±16 ms in Experiment 3b. No significant differences were found between JND values (*p* >0.1).

Overall, we do not find that irregularity in the properties of isochronous fillers leads to a difference in perceived duration. Therefore, the effect of isochronous and anisochronous fillers on duration judgments seems to be specific to irregularity in time and cannot be explained via a general effect of filler predictability or novelty.

## General discussion

The present experiments aimed at investigating the role of the temporal structure of interval fillers on perceived duration. Specifically, intervals with regularly spaced (isochronous) fillers were compared with intervals with irregularly spaced (anisochronous) fillers. Consistent with early reports (Grimm, 1934; Thomas & Brown, 1974), we find that isochronous intervals are perceived as being longer than their anisochronous counterparts, an effect that increases with the level of anisochrony and with the number of fillers.^1^ Our results expand the findings of Thomas and Brown (1974) obtained with a reproduction task by showing that with a direct comparison between isochronous and anisochronous intervals there is a consistent difference in perceived duration; that is, the isochronous interval is perceived as being longer. Such distortions in perceived duration are *not* replicated with fillers that are isochronous but irregular in terms of nontemporal properties (amplitude and frequency). This demonstrates the special role of isochrony of filler signals in the estimation of interval duration. It therefore strengthens our understanding of the filled duration illusion, indicating that what is important is not the characteristics of interval fillers, but *when* those fillers appear.

In addition, we should consider that the two nontemporal irregularity conditions (amplitude and frequency) might as well lead to a deviation from perceived isochrony. It has been shown that the perceptual latency of 1000 Hz sounds measured through simple reaction times varies in a range of roughly 70 ms with a change in stimulus intensity between 40 dB and 80 dB as the one used in Experiment 3a (Pfingst, Hienz, Kimm, & Miller, 1975, as in Luce 1986). For the frequency changes used in Experiment 3b, changes in perceptual latency are roughly 50 ms and have been suggested to be due to the different perceived amplitude that stimuli of a different sound frequency have (Pfingst et al. 1975). According to these values, jittering the fillers’ properties should be perceptually equivalent to presenting them with an anisochrony in the middle-low range of anisochronies used in Experiment 1. The level of perceived anisochrony due to filler properties is thus insufficient to produce a significant difference in perceived duration.

Two contemporary types of models of temporal perception, *interval models* and *entrainment models*, conceive duration estimates to be based on the comparison of sensory information to a memory component. This memory component could either be a duration reference memory as proposed by interval models or the phase and period of the rhythmic context as proposed by entrainment models. In the following, we will take a closer look at the predictions of these models regarding the present data.

### Interval models

Interval models propose a way of representing the duration of an interval via a resettable accumulator-counter mechanism. The internal clock model by Treisman (1963) and the SET model (e.g., Church, Meck, & Gibbon, 1994; Gibbon, 1977; Gibbon & Church, 1990) are prominent examples of such type of models. Previous studies on distortions of perceived duration due to stimulus irregularity have found that unexpected, irregular stimuli in a sequence (oddballs) lead to an overestimation of perceived duration (e.g., Birngruber, Schröter, & Ulrich, 2014; Pariyadath & Eagleman, 2007; Schindel, Rowlands, & Arnolds, 2011). This effect has been explained in the framework of interval models, suggesting that the clock mechanism is sped up by novelty, unpredictability, and irregularity in a sequence. Indeed, it has been shown repeatedly that an increase in arousal or attention due to a stimulus leads to an overestimation of perceived duration (e.g., Burle & Casini, 2001). According to these observations, interval models should predict that (1) irregular intervals should be perceived to last longer than regular ones and (2) such effects should be independent of the type of irregularity (temporal properties or other nontemporal fillers characteristics). Our results however falsify both predictions as filler anisochrony leads to a decrease (rather than an increase) in perceived duration and distortions are observed only for irregularity in time and not in other properties of the fillers.

We should consider, however, that there is a fundamental difference between the current paradigm and the ones in the literature that found an increase of perceived duration with stimulus irregularity. In our study, sequences where either completely regular or completely irregular, whereas the previous results have been obtained from a violation of expectations. For the irregular stimuli of the current experiment, no expectations about stimulus timing (Experiment 1 and 2) or stimulus characteristics (Experiment 3) could be built up. Therefore, it is not surprising that complete interval irregularity does not lead to the arousal/attention effects that have been found in previous studies as no expectations have been violated.

Interval models could in principle account for the current results without appealing to a change in the clock speed if specific characteristics of the clock could explain why isochronous sequences would lead to a higher accumulated duration estimate than anisochronous sequences. This is possible, when assuming (a) a logarithmic relationship between physical and perceived duration (i.e., a concave relationship according to Thomas and Brown’s scheme, 1974), and (b) a reset of the accumulator-counter mechanism at the beginning of each subinterval. The total duration estimate would then be calculated by adding up the duration of the subinterval estimates (Matthews, 2013; Thomas & Brown, 1974). The logarithmic encoding of perceived time is equivalent to a representation of the duration of the overall interval based on the geometric—rather than arithmetic—mean of the subintervals (e.g., Allan & Gibbon, 1991; Church & Deluty, 1977). Whereas the arithmetic mean of 1 s isochronous and anisochronous intervals would be identical, the geometric mean would be larger for isochronous sequences. This could be the reason for an underestimation of interval duration that is specific to irregularity in time and thus explain the main effect in Experiment 1 as well as the lack of an effect in Experiment 3.

To determine whether a logarithmic interval model predicts the observed decrease in PSE values with an increase in temporal irregularity as well as filler number, we derive its analytical expression. To obtain the PSE values for the conditions in the experiments, we need to determine the physical duration of an isochronous interval *T* ^*i*^ that perceptually matches the duration of the anisochronous interval (*T* ^*a*^ =1000 ms), so that

$$ \psi \left({T}^i\right)=\psi \left({T}^a\right). $$

where *ψ* represents the psychometric function relating the physical stimulus to the internal representation, which we assume to be logarithmic. After applying such transformation, the contribution of each of the *N* subintervals (*D* ^*i*^_*s*_ and *D* ^*a*^_*s*_) could be summed to determine the perceived duration of the overall interval at PSE:

$$ {\displaystyle \sum_{s=1}^N} \log \left({D}^i\right) = {\displaystyle \sum_{s=1}^N} \log \left({D}_s^a\right). $$

The anisochronous interval as the standard D^a^ adds up to 1000 ms. The duration of the isochronous interval *T* ^*i*^ is not fixed. The value of D^i^ can be obtained by *D* ^*i*^ *=T* ^*i*^ */N* and substituted in the formula above so that the left-hand side is simplified to:

$$ \log \left({\frac{T}{N}}^i\right)N = {\displaystyle \sum_{s=1}^N} \log \left({D}_s^a\right). $$

From this, *T* ^*i*^ can be obtained analytically according to

$$ {T}^i=N{e}^{\frac{1}{N}}\ {\displaystyle \sum_{s=1}^N}{D}_s^a. $$

**Fig. 5:**
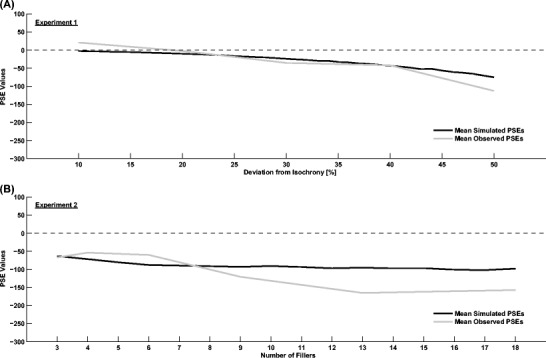
Comparison of the experimental and simulated PSE values (mean over 1000 repetitions for each condition) assuming a logarithmic relationship between physical and perceived time and a clock reset at the beginning of every subinterval. **a** Simulation of Experiment 1. The x-axis represents the deviation of the anisochronous interval from isochrony. **b** Simulation of Experiment 2. The x-axis represents the number of filler stimuli in both the isochronous and the anisochronous interval

The PSE is then simply *PSE=T* ^*i*^ *-T* ^*a*^=*T* ^*i*^ *-*1000. Figure 5 shows the outcome of simulating Experiment 1 and 2, by randomly drawing 1000 samples of an anisochronous interval for each condition and calculating the mean over the respective PSE values. It can be seen that the simulated PSEs follow a pattern similar to the average values obtained experimentally (from Figs. 2 and 3). This similarity confirms that a logarithmic interval model may account for our data in both experiments.

### Entrainment models

Entrainment models (e.g., McAuley & Jones, 2003) explain temporal perception without assuming a resettable clock. They propose perceived duration to be based on oscillatory mechanisms. The peak of the oscillation coincides with the expected time point of stimulus arrival and duration is to be determined in comparison to this point (early or late onset). Phase and period of the oscillation gradually adapt entraining to stimulus sequences. Indeed, effects of neural entrainment to rhythmic sequences have been found in multiple electrophysiological studies. For example, low-frequency oscillations in the primary auditory as well as in the primary visual cortex were observed to adapt their phase to rhythmic stimulus input (e.g., Lakatos, Karmos, Mehta, Ulbert, & Schroeder, 2008; Lakatos, Chen, O’Connell, Mills, & Schroeder, 2007). Neural entrainment at higher frequency bands has been proposed to be the basis of rhythmic perception (e.g., Ding, Sperling & Srinivasan, 2006; Lakatos et al., 2005; Zanto, Snyder & Large, 2006). The peak of the oscillation has been shown to relate to heightened attention and higher neural excitability (e.g., Sanchez-Vives & McCormick, 2000; Steriade, Nunez & Amzica, 1993). That is, the time at which an input arrives will determine whether the input is being amplified or attenuated depending on the phase of the underlying neural oscillation. In this sense, entrainment has been suggested as a mechanism of attentional selection, changing response gain and reaction times with an expected stimulus (e.g., Cravo, Rohenkohl, Wyart, & Nobre, 2013; Fries, Schröder, Roelfseman, Singer, & Engel, 2002; Lakatos et al., 2008; Schroeder & Lakatos, 2009). Following this idea, fillers of a regularly spaced interval would likely coincide with the peak of the entrained oscillatory period, that is, the point of highest neural excitability.

It has been suggested that perceived duration increases with an increase in neural response towards a stimulus (e.g., Eagleman & Pariyadath, 2009). This does not only give a framework to explain effects of arousal and attention (e.g., Thomas & Weaver, 1975; Burle & Casini, 2001), but it also can account for the filled duration illusion as filled intervals should have an increased neural response compared with empty ones (e.g. Thomas & Brown, 1974; Wearden, Norton, Martin, & Oliver, 2007) and the increase is a function of the number and duration of the fillers (e.g., Buffardi, 1971). Assuming that the neural response towards fillers is strongest at the beginning of an interval and habituates with repeated exposure (e.g., Polich, 1989) also the finding of a higher impact of stimuli in the beginning compared with the end (e.g., Buffardi, 1971; Adams, 1977) conforms to the idea of a link between perceived duration and neural response magnitude.

It is not immediately evident why isochronous intervals would elicit higher responses and an increase in perceived duration as compared to anisochronous ones, given that the total magnitude of the sensory input is identical. In the framework of neural entrainment, however, an isochronous sequence causes fillers to arrive at the peak of entrained neural oscillations, leading to amplification and thus to higher overall neural activity. On the other hand, fillers in an anisochronous sequence are unlikely to arrive at the same phase of the neural oscillation, thus causing different (and lower) levels of amplification. This leads to a lower overall neural response to the fillers in an anisochronous interval when compared to an isochronous interval. Therefore, perceived duration, if it is related to neural response magnitudes, should be longer for isochronous than for anisochronous sequences as observed in Experiment 1. The account of entrainment related to neural response magnitudes would as well predict the results of Experiment 2. Assuming predictability and thereby neural entrainment to built up with the number of isochronous stimuli (e.g., Stefanics et al., 2010), an increased number of fillers leads to an increased response towards isochronous in contrast to anisochronous stimuli. This may explain our finding of an increase in the difference between perceived isochronous and anisochronous duration with an increase in the number of fillers. Finally, isochronous fillers that are irregular for nontemporal properties would entrain the neural oscillation in the same way as regular fillers do. In accordance to this prediction we find no difference in perceived duration due to nontemporal irregularity in Experiment 3.

## Conclusions

Our results demonstrate longer perceived duration estimates due to regularity in time (isochrony) compared with temporal irregularly (anisochrony). Such a bias in perceived duration is not present when nontemporal properties of the fillers are made irregular. We show that the change in perceived duration as a function of anisochrony level and number of stimuli is consistent with a logarithmic encoding of perceived duration in the framework of a resettable clock (Thomas & Brown, 1974; Matthews, 2013). On the other hand, the perceptual difference between isochronous and anisochronous intervals could be explained in the context of entrainment models, because isochronous filler stimuli coincide with higher neural excitability and lead to increased magnitude of the overall neural response. As the entrainment increases with more filler stimuli, the response gain becomes larger and the difference in perceived duration between isochronous and anisochronous sequences becomes more evident. Simulations confirm that the observed distortions of perceived interval duration due to temporal structure are in accordance with the predictions of a logarithmic interval model. In order to determine whether the predictions of the entrainment model are quantitatively consistent with our results, we would need to identify the function relating neural response magnitudes to perceived duration, which at the moment is unknown.

## Footnotes

1 To make sure that the observed difference in perceived duration is not due to the repeated presentation of multiple trials, but can already be found in a single comparison, we asked 60 participants to make a single 2IFC judgment in the manner of Experiment 1. We used the highest level of anisochrony and the number of fillers from Experiment 1 (5 signals for each interval). The order of the two one-second intervals was counterbalanced between participants. 76.7 % of participants judged the isochronous interval to be longer (χ^2^(1)=21.7, p<0.001), demonstrating that the difference in perceived duration between isochronous and anisochronous intervals is present already at individual trial level.
